# New Orientation of Study on Economic Psychology and Behaviour

**DOI:** 10.1515/tnsci-2019-0015

**Published:** 2019-05-21

**Authors:** Yucong You

**Affiliations:** 1 Guangzhou College of Technology and Business, Guangzhou 510000,China

**Keywords:** Economics, behavioral economics, economic decision-making, psychological behavior, virtual economy

## Abstract

Economic psychology refers to the impact of psychological factors on economic changes, and its outward manifestation is economic behaviour. Psychology, as a science studying human psychology and behaviour, has no reason to ignore the study of economic activities. This study summarizes the latest research results and conclusions of economic psychology from three aspects of behaviour level, body signal and other people’s movement or displacement. In addition, it expounds the reflection of economic psychology, the reflection of rational human hypothesis of traditional economics and the prospect of future research. From the perspective of economic psychology, it is of great significance to analyse the psychological motivation behind the conflicts and interests in the study of economic psychology and behaviour so as to construct the harmonious behaviour relationship of “psychological contract” on the basis of the rational mechanism of interest distribution.

## 1 Introduction

Economic psychology is a science that studies the psychological reflection of people’s production relations, economic policies and economic mechanisms, also known as psych-economics. The economic psychology was born in the early 1950s and was formed by a group of “economists with good psychological literacy” and “psychologists with good economics.”

For a long time, traditional economics has focused on the formal expression of mathematics and tried to avoid subjective analysis, and used this as a means of distinguishing between sociology and psychology. A typical reflection of this tendency is to exclude the influence of the body and its dynamics from the economic theory system. In fact, the notion that physical factors “interfere” with reason can be traced back to Plato’s thoughts about innate ideas. He believes that physical experience helps reveal some of the innate knowledge, but it also distorts this knowledge. For example, in the “Many Pieces” Plato pointed out: “... eyes, ears and even the whole body are interfering factors that hinder the soul’s acquisition of knowledge.” For rational economists, this irrationality the factors are to be eliminated, so it is often psychologists rather than economists who have long focused on physical factors and their effects on behaviour. Because economics and various decision-making models avoid the potential physiological mechanisms, most of the neuroscience and biological variables have nothing to do with economic theory verification; some economists are aware of the physical factors. Knowing the impact of the activity, but still refusing to incorporate it into economic theory, such as the human behavioural view of the Austrian economist’s representative, Mises, that economics should only study “willingness” and “intentional behaviour”: “economics are not considered ... part of the body’s organs and instinct’s passive response to stimuli, because it is not controlled by the will.” These views and derived behaviours are mutually causal, which together lead to traditional economics that have long ignored the physical factors of behavioural subjects for decision-making, the influence of economic behaviour such as preference has become an economic theory of “away from”.

It uses psychological perspectives to analyse the psychological factors in the economic process, and takes the decision-making process of consumers and enterprises’ economic behaviours as well as the psychological factors that influence these decision-making processes. The basic task is to explore how consumers, entrepreneurs, and political decision-makers will perform under different conditions, and what decisions will be made to understand and predict the economic processes that will occur in specific situations. . Its research content includes: investigating and analysing psychological factors of people’s economic behaviour, such as needs, motivation, attitude, willingness, expectation and other psychological conditions; investigating and studying the behaviour of consumers and corporate decision-making figures, analysing and comparing the economy under different circumstances behaviour, and generalization; focus on behavioural decision-making processes related to consumption, savings, investment, and the like.

**Figure 1:**
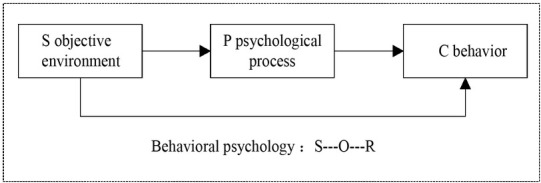
Katona’s Economic Psychology Model

With the deepening of the study on experimental psychology, psychologists apply its knowledge to the concrete analysis of the surrounding problems, which not only cover personal thought, behaviour, perception and emotion, but also include group behaviours ^[1]^. The economic psychology model of Katona is shown in Figure 1. Among humanism and cognitive psychology, behaviourism, psychological analysis, Gestalt, and cognitive psychology have a greater impact on economics ^[2]^.

### 2 The change of psychology and its main genre

In the economics community, Herbert Simon and Friedrich Hayek, who first called for the inclusion of physical factors in economic analysis, presented a series of forward-looking perspectives that inspired the development of embodied economics. Simon explores the decisive and important role of the body (sensory and motivational systems) in behaviour, emotion, and decision making. Simon believes that emotions can delay judgment, allow individuals to sort information, and intercept the information necessary for decision making. Hayek, the representative of neoclassical economics, was one of the first economists to advocate that economics should draw on the results of psychological research. He discussed in the discussion of the issue of anthropomorphism: “We will anthropomorphize what we observe.” The tendency may be a result of the use of schemas that are provided by our own physical movements.” This concept emphasizes the connection between the individual itself and the “causality” of the world’s perceptions of the world and provides a handle for understanding the world of redundant information.

Since 1879, the entire psychology community has experienced a prosperous academic discussion that has never been seen before. After the content psychology of Feng Te, another two-phase three-phase succession or opposition or the inheritance theory of Feng Te, or another path, unique. There are hundreds of psychology classes of all kinds, large and small. These schools are widely distributed throughout the world. All schools, including the schools of mutual inheritance, are related and different in their psychological research objects, scope, nature, content, and methods. The speed and research results of the development of psychology over the past 100 years far exceed all the sums of the results of psychological research in human history, and the depth and breadth of the exploration of psychological phenomena have reached an unprecedented level.

**Figure 2:**
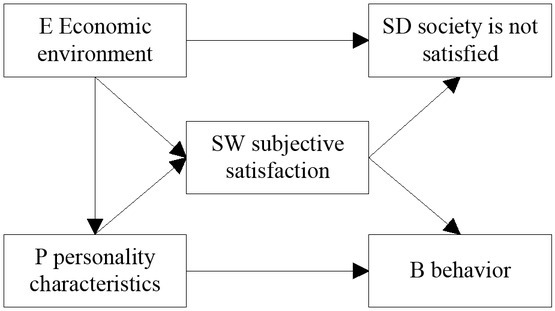
Sturm Bell’s extended model

“Capital is not a matter but a kind of social relation” proposed by Marx seems to be easier to be understood than the era of industrialization. The expansion of the value form of capital is combined with the informational economy so as to increase the circulation speed of capital and expand scale. As the stock form of capital, the price of assets is no longer only related to physical assets. The expansion model of Sterm Bell is shown in Figure 2 ^[3]^.

**Figure 3:**
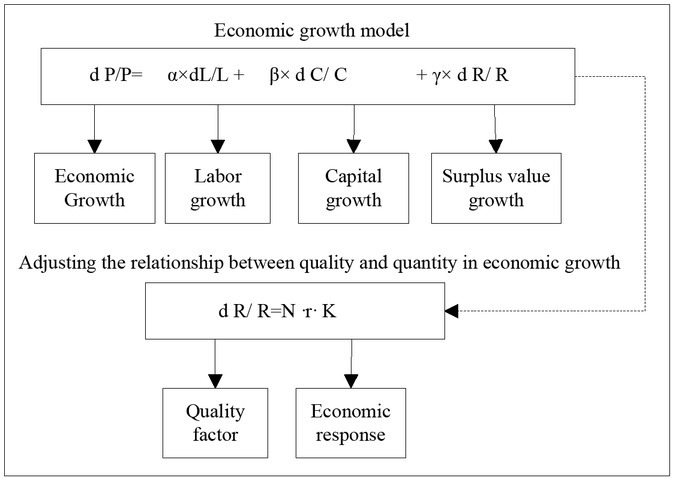
Renault’s overall model

Economics and all kinds of decision-making models avoid talking about the underlying physiological mechanism, so most of the neuroscience and biological variables have nothing to do with the verification of economics theory. In recent years, the development of economic geography has mainly focused on the basic research field and especially paid special attention to the explanation and research of some anomalies contrary to the traditional economic law. There are a lot of small points of view, such as Renault’s overall model (as shown in Figure 3). Although study of its application area is relatively scarce, there are some developments and most studies are about consumption area.

## 3 The path of physical factors affecting economic decision-making

The behavioural factor is multi-dimensional. The perceptual symbol theory emphasizes that the process of processing knowledge information includes two states. One is the real body state and the other is the psychological state which simulates the experience (the individual’s body and the interaction between body and environment provide the prototype for the individual to know the world) ^[4]^. It can be found from the empirical study of economics that there are possible paths for the impact of physical factors on economic decision-making.

### 3.1 Advances in cross-cultural research on individual judgment and decision making

Although behavioural economics insists on subjective axiology and rational hypothesis, it has gradually become an independent school to appear in the jungle of contemporary economics through the challenge to rational-economic man himself and the use of psychology to construct its own behavioural basis. The hard core of behavioural economics can be compared with that of neoclassical economics, as shown in Table 1:

**Table 1:** Comparison of Behavioural Economics and Neoclassical Economics

Category	Hard core	Protective tape	Research methods
Neoclassical	Rational economic man assumes;	Equilibrium; marginal utility or	Methodological marginal analysis
economics			method;
	Preference and endowment	Free movement of elements and products;	Individualism;
	Distribution exogenous	Production-homogeneity of elements and products	Static and comparative static analysis
	Subjective value theory;	diminishing;	
	Transaction relationship as center	Price recipients,	
Behavioural economics	Bounded rationality party assumptions	Unbalanced	Methodological individualism;;
	Possible pursuit of altruistic behavior and irrational behavior	Nonlinear utility function	Evolution analysis;
	Preference and endowment endogenous	Heterogeneity of elements and products	Nonlinear programming
	Learning process; subjective value theory	Randomness	Experimental and microscopic
			measurement

### 3.2 The impact of comparative research on decision bias and rational mechanism

To compare the extent to which economic volume in different economic entities are affected by changes in certain factors, “economic entity elasticity” is used as a tool ^[5]^. Economic entity elasticity indicates the extent to which economic volume responds to changes in certain influence factors. Expressed by the formula, it is the ratio of the change rate of economic volume to the change rate of influence factors, as shown in Formula (1) ^[6]^.

(1) $$E=\frac{\Delta Q\%}{\Delta X\%}$$

Where, E is economic entity elasticity; Q is economic volume; X is a certain factor affecting economic volume. The price elasticity of an economic entity reflects the extent to which the economic volume reacts to price change. In other words, one percent of change in price will cause a few percent of change in the economic volume. The calculation formula can be calculated by Formula (2):

(2) $$o\left(w\right)=d_{1}∙d_{2}∙\left(\frac{1}{n}\sum_{i}sim\left(w,\;p_{i}\right)-\frac{1}{m}\sum_{j}sim\left(w,\;n_{j}\right)\right)$$

Where, Q is economic volume; Δ*Q* is absolute quantity of economic volume change; P is price; Δ*P* is absolute quantity of price change. The elasticity of a point on the demand curve (the quantity of Δ*Q* and Δ*P* is as small as 0) and can be calculated by Formula (3))

(3) $$\varepsilon_{p}=\underset{\Delta p\to 0}{\lim}\frac{\Delta Q}{\Delta P}∙\frac{P}{Q}=\frac{dQ}{dP}∙\frac{P}{Q}$$

Where, ε *_p_* is price elasticity of a certain point. The point elasticity is calculated on the premise that the equation of the demand curve must be known.

There is a gambling game ( *p*, *x*;*q*, *y*) , where *p* + *q* <1 and *x*, *y*∈*R* . Behavioural economics believes parties seek to maximize v a l u e *U* ( *p*, *x*;*q*, *y*) = π (*p*)*v*(*x*) + π (*q*)*v*( *y*) . Due to the non-linearity of the probability weight function caused by the risk attitude of the party and the non-linearity of the value function of the party, the decision-making is faced with various possible combinations, as shown in Table 2 below:

**Table 2:** Expectation risk attitude and behavioural characteristics

	Small probability	Medium and high probability
income	Risk hobby	Risk avoidance
loss	Risk avoidance	Risk hobby

If the time factor is considered in decision-making, behavioural economists find that the discount utility model that neoclassical economics relies on in inter-temporal decision-making also lacks a scientific basis, which can be expressed by Formula (4).

(4) $$U^{t}\left(c_{t},\ldots ,c_{T}\right)=\sum_{k=0}^{T-t}D\left(k\right)u\left(c_{t+k}\right)$$

$D\left(k\right)={\left\{\frac{1}{1+\rho }\right\}}^{k}$. In the theory of discount utility, the inter-temporal preference of the decision-maker to the consumption bundle ( *c_t_* ,..., *c _T_* ) can be expressed by the above-mentioned discount utility function. What the decision-maker needs to do is to estimate the utility flow of each period in the future, and then converts it into the present value through a unified discount rate ρ so that the static utility maximization problem is transformed into the dynamic utility present value maximization problem.

### 3.3 Behavioral Research and Economics and Macroeconomic Policy Analysis

**Figure 4:**
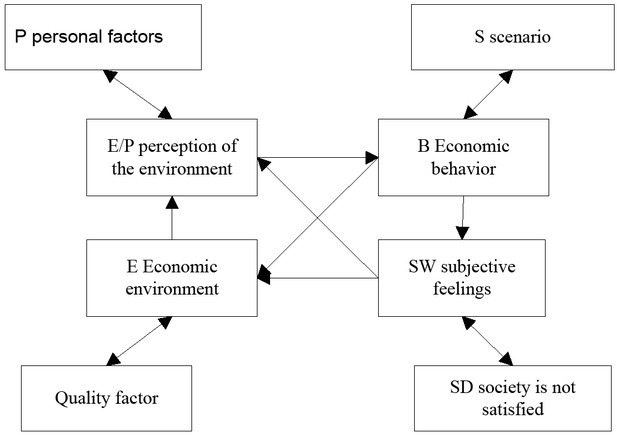
Feedback between economic behaviour and personal personality characteristics

Behavioural economics is the branch of economics that studies economic behaviour and economic phenomena on the basis of psychology. Its core point of view is that the study of economic behaviour must be based on the actual psychological characteristics instead of the abstract behaviour hypothesis [7]. Economic psychology is a science that studies the relationship between the mental world of individuals and groups and the efficiency of economic activities. As a result, market effectiveness is no longer established and various economic policies need to be reconsidered, as shown in Figure 4 ^[8]^.

**Figure 5:**
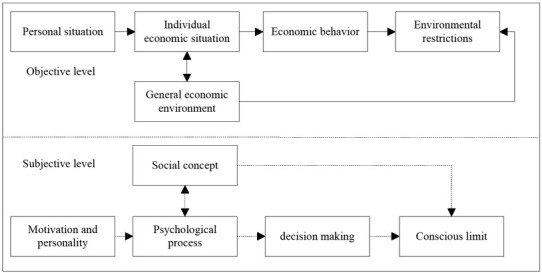
The role of subjective and objective behavioural factors in economic behaviour

Economists have realized economic anomalies and slowly accepted these irregularities. It is the development of experiment-based psychological analysis that proves that this is a new and promising direction. Finally, the comprehensive application of research technology of economic psychology to psychology and economics can better explain and predict the economic behaviour of human beings. Psychology makes it possible to validate and construct theories that logic deductions in economics can’t do by testing the theoretical framework with a small sample, as shown in Figure 5.

## 4 Achievements and Prospects of Behavioral Decision-Making in Economic Psychology

Although economists always ignore the existence of psychology, they have to consider psychology and have no economics that completely gets rid of psychology. For example, according to the classical proposition “Human behaviour is mechanical” of the behaviourism psychology school, economics puts forward the hypothesis that man is a machine, and the relationship between man and objective things is a simple stimulus-response (S-R). On this basis, “the function that consumption expenditure is income” and “the function

that investment is profit” are constructed. In addition, the economic principles and even the whole theoretical system of traditional economics, such as no increase in demand for decrease in price, no decrease in demand for increase in price are constructed.

### 4.1 Physiological status and economic behavior

Traditional economics doesn’t pay much attention to the impact of physiological state on economic decision-making, but the studies of physiological psychology and neuro-economics have found that physiological clues such as metabolism and hormone level can directly affect individual’s preference and choice in decision-making [9]. The dynamic adjustment of risk preference depends on three aspects: metabolic status, energy reserve and uptake rate. That the energy uptake rate is lower than the reference point will lead to greater risk seeking for the individual. However, that the energy uptake rate is higher than the reference point will lead to greater risk aversion [10]. The role of psychological factors in economic behaviour is shown in Figure 6.

As shown in Figure 6, the metabolic reference point is usually the uptake rate required for reaching the survival threshold. When the uptake rate is lower than the threshold, the probability of starvation will increase to promote the behaviour of risk seeking. 200 lottery tickets of different risk levels are presented to the subjects for selection. At the same time, the levels of leptin and ghrelin are measured. It is found that the change of risk preference is positively correlated with the baseline levels of leptin and ghrelin in individuals ^[11]^.

### 4.2 The Acquisition and Application of Behavioral Research Results and Methods in Economic Psychology

When people make decisions by using physical information, they are sometimes aware of the existence of these information clues, but sometimes they are not. According to the above mapping method of the regulatory effect, the regulatory effect of trust to behaviour on the relationship between behaviour mobilization behaviour and expectation of change prospect is shown in Table 3. It shows that when the trust of behaviour is at a high level, the behaviour mobilization behaviour has a more positive effect on the expectation of change prospect.

The model MD0 and the model MD1 in Table 3 examine whether the trust to behaviour regulates the positive relationship between the behaviour mobilization behaviour and the change atmosphere perception by taking the change atmosphere perception as a dependent variable. Model MD0 (F = 27.67, P < 0.001) and MD1 (F = 31.71, P < 0.001) have significant explanatory power. In model MD0, the behaviour mobilization behaviour has a significant positive effect on the change atmosphere perception (β = 0.37, P < 0.001), which supports the hypothesis H6. The interaction items of behaviour mobilization behaviour and trust behaviour are added to model MD1, which increases the R–square of the model by 0.06 (P < 0.05). The regression coefficient of the interaction items is significantly positive (β = 0.14, P < 0.05).

**Table 3:** Adjustment effect test result

Dependent variable	Expectation of change prospects		Change atmosphere perception		Change effectiveness perception	
Model number	MC0	MC1	MD0	MD1	ME0	ME1
Working age factor	-0.02	0.01	0.02	0.03	0.03	0.03
gender	-0.02	-0.01	-0.03	-0.02	-0.02	-0.02
Education level	0.03	0.06	0.05	0.05	0.05	0.04
department	-0.05	-0.05	-0.05	0.02	0.02	0.01
Leadership mobilization	0.41***	0.42***	0.36***	0.27**	0.54***	0.53***

## 5 Conclusions

A large number of studies and theories of economic psychology and behaviour need to be sorted out and integrated. Although lots of studies have proved the impact of somatization factor on economic behaviour such as preference, judgment, valuation, consumption, decision-making and risk perception, and a series of new theories with domain characteristics have formed, the researches on decision-making and judgment in behavioural economics make the boundary between psychology and economics become fuzzy. Neuro-economics makes people begin to pay attention to the connections between the neurobiological basics of economic behaviour. Besides, we may realize that initiating paradigm researches may have problems. Many behavioural studies of economic psychology and behaviour basically adopt the initiating paradigm. If the expanded scope of economic science is not recognized or if people don’t actually apply this knowledge, it is possible to initiate different symbolic representations or metaphors for the same body clues even if different modes of thinking under different cultures. Taking the touch study as an example, numerous studies have found that touch can lead to more generous economic behaviour.

**Figure 7:**
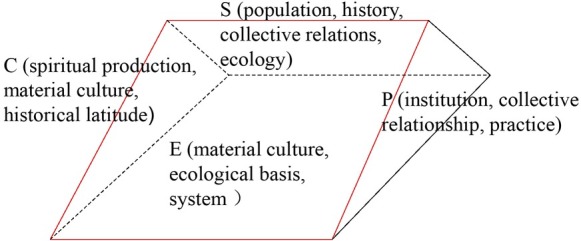
Role of psychological factors in economic behaviour
